# Macrophage environment turns otherwise MccJ25-resistant *Salmonella* into sensitive

**DOI:** 10.1186/1471-2180-13-95

**Published:** 2013-05-01

**Authors:** María Fernanda Pomares, Natalia S Corbalán, Conrado Adler, Ricardo de Cristóbal, Ricardo N Farías, Mónica A Delgado, Paula A Vincent

**Affiliations:** 1 Instituto Superior de Investigaciones Biológicas (Concejo Nacional de Investigación Científica y Técnica-Universidad Nacional de Tucumán) and Instituto de Química Biológica “Dr. Bernabe Bloj”, Chacabuco 461, San Miguel de Tucumán 4000 Tucumán, Argentina

**Keywords:** Microcin J25, *Salmonella* Typhimurium, FhuA, Low pH

## Abstract

**Background:**

Microcin J25 (MccJ25) is a plasmid-encoded antibiotic peptide produced by *Escherichia coli* (*E. coli*). MccJ25 enters into the sensitive *E. coli* strains by the outer membrane receptor FhuA and the inner membrane proteins TonB, ExbB, ExbD and SbmA. The resistance of *Salmonella enterica* serovar Typhimurium (*S.* Typhimurium) to MccJ25 is attributed to the inability of its FhuA protein to incorporate the antibiotic into the cell.

**Results:**

In this work we demonstrate that *S.* Typhimurium becomes notably susceptible to MccJ25 when replicating within macrophages. In order to determine the possible cause of this phenomenon, we studied the sensitivity of *S.* Typhimurium to MccJ25 at conditions resembling those of the internal macrophage environment, such as low pH, low magnesium and iron deprivation. We observed that the strain was only sensitive to the antibiotic at low pH, leading us to attribute the bacterial sensitization to this condition. A MccJ25-resistant *E. coli* strain in which *fhuA* is deleted was also inhibited by the antibiotic at low pH. Then, we could assume that the MccJ25 sensitivity change observed in both *E. coli fhuA* and *S.* Typhimurium is mediated by a MccJ25 uptake independent of the FhuA receptor. Moreover, low pH incubation also sensitized *S.* Typhimurium to the hydrophobic antibiotic novobiocin, which does not affect enteric bacteria viability because it is unable to penetrate the bacterial outer membrane. This observation supports our hypothesis about low pH producing a modification in the bacterial membrane permeability that allows an unspecific MccJ25 uptake. On the other hand, MccJ25 inhibited *S.* Typhimurium when cells were preincubated in acidic pH medium and then treated at neutral pH with the antibiotic.

**Conclusions:**

Our results suggest that acidic condition does not alter MccJ25 hydrophobicity but irreversibly modifies bacterial membrane permeability. This would allow an unspecific antibiotic uptake into the cell.

From our data it is possible to infer that intracellular pathogenic strains, which are *in vitro* resistant to MccJ25, could become susceptible ones *in vivo.* Therefore, the MccJ25 action spectrum would be broader than what *in vitro* experiments indicate.

## Background

Microcin J25 (MccJ25) is a 2,107-Da peptide antibiotic which is constituted by 21 unmodified amino acids and is excreted to the culture medium by *E. coli* strains harboring the MccJ25-coding plasmid [1,2]. Uptake of this antibiotic into *E. coli* is dependent on the outer-membrane receptor FhuA [3] and the inner membrane proteins TonB, ExbB, ExbD, and SbmA [4]. Energy provided by the proton motive force of the cytoplasmic membrane and the TonB–ExbB–ExbD protein complex is required for active transport through FhuA [5]. Once inside the sensitive cell, the peptide is able to inhibit *E. coli* RNA polymerase (RNAP) and the membrane respiratory chain [6-8].

This antibiotic is active against bacteria related to the producer strain such as *Salmonella*, *Shigella* and *E. coli*, while other *Enterobacteriaceae* are resistant [9]. Then, it is possible to say that MccJ25 shows, *in vitro*, a narrow action spectrum. Currently, we are interested in MccJ25 action on *Salmonella*, a facultative intracellular pathogen responsible for a variety of diseases in a wide range of animal species. In humans, this pathogen may cause gastroenteritis (food poisoning), septicemia and typhoid fever. Several *Salmonella enterica* strains showed high sensitivity to MccJ25, while others like *S.* Typhimurium, *S.* Derby, and some *S*. Enteritidis strains were completely resistant [9]. Since, transforming resistant *Salmonella* strains with a plasmid coding for the *E. coli fhuA* gene rendered them hypersensitive to the antibiotic, we concluded that the intrinsic resistance is due to the inability of the *Salmonella* FhuA protein to mediate the MccJ25 uptake [9]. In fact, MccJ25 was able to inhibit both intracellular targets in the resistant *Salmonella* strains carrying *E. coli fhuA*[9]. Based on these results it was postulated that the outer membrane is the principal barrier that MccJ25 has to overcome to reach its targets.

Recently, we demonstrated that the membrane permeabilizing peptide, (KFF)_3_K, allows the MccJ25 uptake independently of FhuA and SbmA receptors thus turning microcin naturally resistant strains into susceptible ones [10]. Moreover, the same effect of (KFF)_3_K on *S.* Typhimurium susceptibility to MccJ25 was observed in bacteria replicating within eukaryotic cells. Furthermore, an interesting observation was that MccJ25 itself was able to inhibit 30% of the *S.* Typhimurium intracellular replication [10]. The goal of the present study was to address the mechanism causing this phenomenon. Our data demonstrate that the low pH affects the bacterial membrane permeability *in vitro*, indicating that this mechanism could be also responsible for the *S.* Typhimurium sensitization once it is phagocytized and transferred to vacuoles.

## Results and discussion

### Effect of macrophages internal environment on *S.* Typhimurium sensitivity to MccJ25

**Figure 1:**
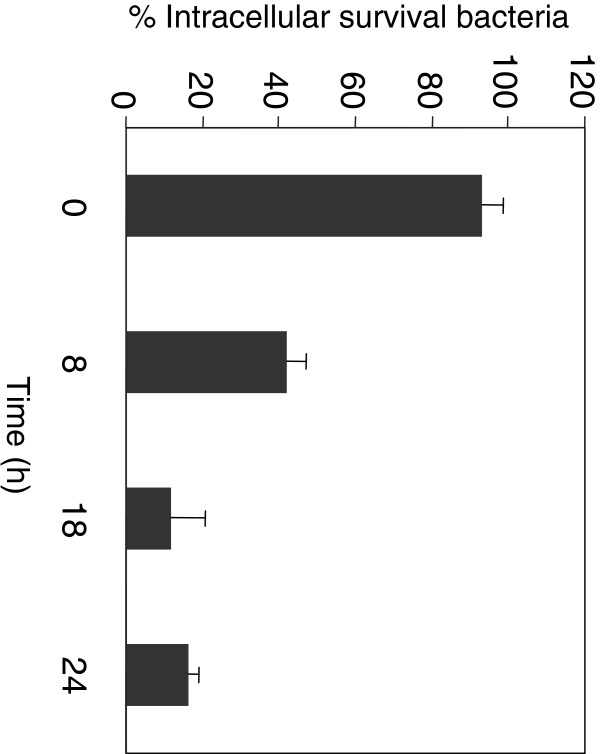
**Effect of MccJ25 on *S. *Typhimurium intracellular replication.** RAW 264.7 macrophages were seeded in 24-well plates and grown for 24 h before bacterial infection with an overnight culture of *S.* Typhimurium 14028s strain. The infected macrophages were treated with MccJ25 (117.5 μM) and the number of viable intracellular bacteria was determined at 0, 8, 18 and 24 h post-treatment. Values are presented as the percentage of intracellular surviving bacteria (CFU mL^-1^) recovered from macrophages treated with MccJ25 referred to the CFU mL^-1^ obtained from untreated macrophages. Error bars represent standard deviations from five independent experiments.

**Figure 2:**
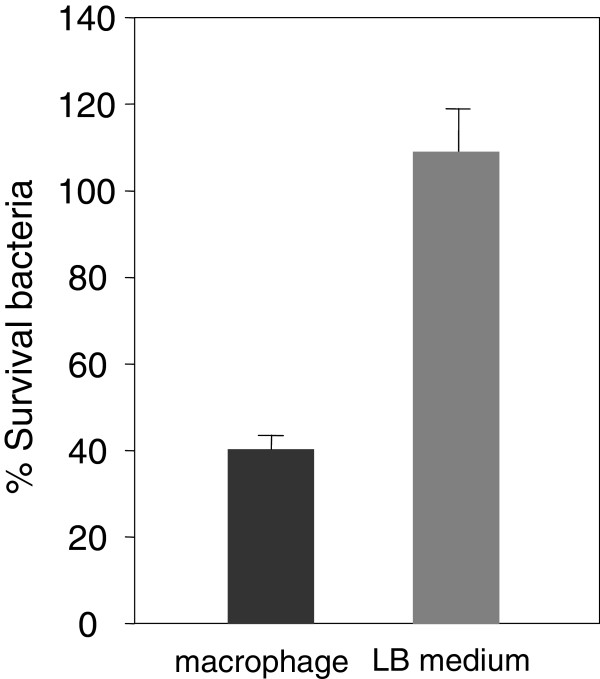
**Effect of macrophage internal environment on *S. *Typhimurium sensitivity to MccJ25.** 10^6^ mL^-1^ bacteria harvested from lysed infected RAW 264.7 macrophages and a bacterial suspension (10^6^ mL^-1^ cells) in 0.2% Triton X-100 obtained from an LB culture were incubated at 37°C for 6 h with or without 117.5 μM MccJ25. Bars represent the percentage of bacteria surviving MccJ25 treatment CFU ml -1 after growing in LB (grey bar) or within macrophages (dark bar). For each condition, the percentage is referred to the CFU mL^-1^ obtained with no addition of MccJ25. Error bars represent standard deviations from five independent experiments.

We previously showed that, although *S.* Typhimurium is a MccJ25-resistant strain *in vitro*, its intracellular replication was moderately inhibited by the antibiotic (about 30%, 6 h after bacteria internalization) [10]. In the present work we observed that the number of surviving *S.* Typhimurium cells within macrophages decreased 60% after 8 h of MccJ25 exposition compared with the control (without MccJ25). This effect was strongly increased with time, reaching between an 80-90% of intracellular replication inhibition after 18 h of MccJ25 treatment (Figure 1). A potential explanation for this effect is an unspecific MccJ25 uptake produced when the pathogen grows within macrophages. In order to prove this hypothesis we determined, *in vitro*, the MccJ25 sensitivity of *S.* Typhimurium cells grown for 8 h within macrophages in the absence of the antibiotic (see Methods). We observed a 58% viability decrease when bacteria directly harvested from macrophages (fraction from lysed macrophages) were incubated with MccJ25 for 6 h, compared with the control (bacteria incubated without antibiotic) (Figure 2, macrophage). Additionally, we determined the MccJ25 sensitivity of bacteria grown on LB medium and resuspended in Triton X-100 (solution used to harvest intracellular bacteria) and no MccJ25 effect on bacterial viability was observed (Figure 2, LB medium).

### Low pH effect on susceptibility of *S*. Typhimurium to MccJ25

**Figure 3:**
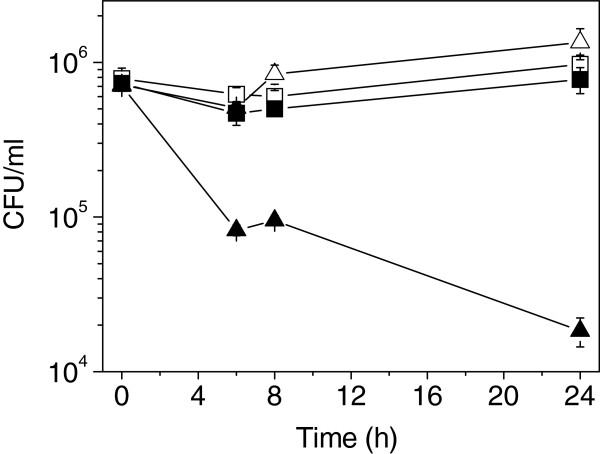
**Effect of low pH on *S. *Typhimurium susceptibility to MccJ25.** 10^6^ mL^-1^ cells of *S.* Typhimurium 14028s strain were incubated at 37°C in M9 medium pH 7 with (black squares) or without (white squares) 117.5 μM MccJ25 and in M9 pH 4.7 in presence (black triangle) or in absence (white triangle) of 117.5 μM MccJ25. At 0, 6, 8 y 24 h post-treatment, the CFU mL^-1^ was determined. Error bars represent standard deviations from five independent experiments.

When bacteria replicate within eukaryotic cells, many changes in the membrane are produced in response to the internal environment. For example, acidic conditions, low magnesium and iron concentrations are some of the host-cell internal conditions to which the bacteria must adapt to [11]. As we observed that MccJ25 affects *in vitro* the viability of *S.* Typhimurium previously replicated within macrophages (Figure 2), we investigated which macrophage environmental condition would allow an unspecific MccJ25 uptake. When bacteria were grown under low magnesium concentration (10 μM) or under iron deprivation (T medium without iron), no changes in MccJ25-resistance was observed (Data not shown). On the contrary, when bacteria were cultured with MccJ25 (117.5 μM) in acidic medium (pH 4.7), the number of CFU mL^-1^ (colony-forming units per milliliter) was 2 orders of magnitude lower than the bacteria grown without the antibiotic, after 24 h (Figure 3). As expected, no antibiotic effect of MccJ25 was observed when pH 7 medium was used in a similar assay (Figure 3).

Furthermore, we studied the effect of low pH on the sensitivity to MccJ25 of a MccJ25-resistant *E. coli* strain. For this, we determined the antibiotic sensitivity of MC4100 *fhuA*::Km strain (mutant in the MccJ25 outer-membrane receptor) in M9 medium plates either at pH 7 or pH 4.7. As expected, this strain became susceptible to the antibiotic at pH 4.7 (MIC = 58.75 μM), while at pH 7, the bacterium was resistant (resistant to 470 μM MccJ25 solution). Since the *fhuA* gene is totally deleted in the MC4100 *fhuA*::Km strain, we could assume that the sensitivity changes observed in both *E. coli fhuA * and *S.* Typhimurium are mediated by an FhuA-independent MccJ25 uptake.

Taken together, our results suggest that low pH could alter the outer membrane permeability letting MccJ25 to reach its intracellular targets and consequently to inhibit the bacterial growth. Furthermore, the high MccJ25 concentration required to inhibit *S.* Typhimurium growth at low pH or within macrophages is indicative of the unspecific nature of the antibiotic uptake. Our interpretation is supported by the observation that a variety of stresses can produce a modification in the outer membrane barrier of Gram-negative bacteria [12-15]. Alakomi *et al.*[16] reported that lactic acid (pH 4) was capable of permeabilizing *E. coli*, *Pseudomonas aeruginosa* and *S.* Typhimurium by disrupting the outer membrane. Thongbai *et al.*[17] proposed that exposure to low pH can alter the outer membrane permeability barrier and allow lethal compounds, normally unable to penetrate, to go through the modified bacterial membrane. In agreement with our data, authors reported that *S*. Typhimurium cells, at pH 4.5, lose the outer membrane integrity allowing cetylpyridinium chloride (CPC)-nisin access to the cytoplasmic membrane which results in the cell death [17].

**Figure 4:**
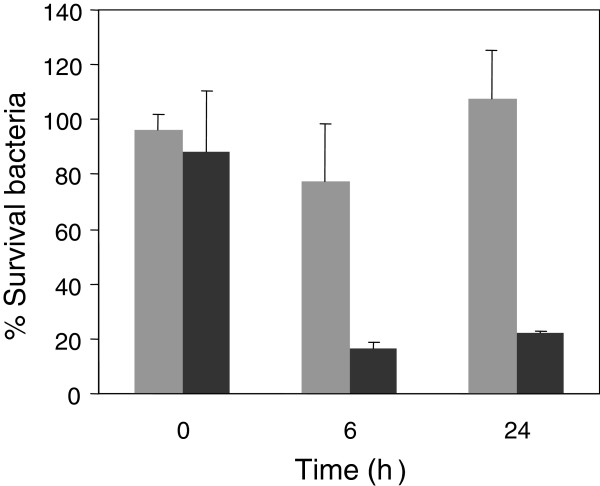
**Effect of low pH preincubation on *S. *Typhimurium sensitivity to MccJ25.** The *S.* Typhimurium 14028s strain was incubated at 37°C during 0, 6 and 24 h in M9 medium pH 7 (grey bars) or pH 4.7 (black bars). At mentioned times, cells were washed, resuspended in PBS and then incubated for 6 h with or without MccJ25 (117.5 μM). Finally, the number of surviving bacteria (CFU mL^-1^) was determined by plating on LB agar. Values are presented as percentage of bacteria (CFU mL^-1^) obtained after MccJ25 treatment referred to the control (with no antibiotic addition). Error bars represent standard deviations from five independent experiments.

Yamaguchi *et al.*[18] showed that the lower the pH of the medium, the higher the accumulation of tetracycline in *E. coli*. In this report, authors concluded that the molecule taken up across the membrane is a protonated form of tetracycline. In this sense, we considered the possibility that MccJ25 could become more hydrophobic under low pH thereby favoring entry into the cell. To rule out this possibility, we performed an assay where only bacteria were exposed to low pH effect. For this, bacteria were previously incubated in M9 medium either at pH 7 or 4.7 for different times, washed with PBS (pH 7.4) and then treated for 6 h with MccJ25 (117.5 μM). As seen in Figure 4, bacteria preincubated for 6 and 24 h at pH 4.7 were susceptible to the antibiotic, while those preincubated at pH 7 remained resistant. These results suggest that low pH makes resistant bacteria susceptible to MccJ25 by significantly changing the bacterial physiology rather than by modifying MccJ25 hydrophobicity.

**Figure 5:**
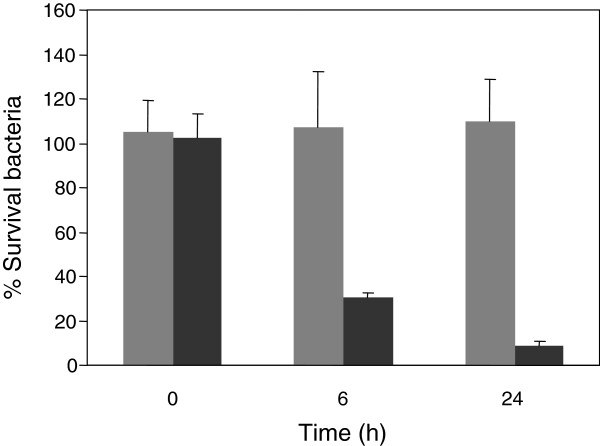
**Effect of low pH on the sensitivity of *S. *Typhimurium to novobiocin.** 10^6^ mL^-1^ cells of *S.* Typhimurium 14028s strain in M9 medium pH 7 (grey bars) or pH 4.7 (black bars) were treated with 0.15 μM novobiocin or sterile bidistilled water as control. CFU mL^-1^ was determined after 0, 6 and 24 h of incubation at 37°C. Results are expressed as percentage of surviving bacteria to novobiocin relative to the control in the absence of the antibiotic. Error bars represent standard deviations from five different experiments.

Ofek *et al.*[19] proposed that resistance to novobiocin in Gram-negative enteric bacteria is probably due to the inability of the antibiotic to penetrate the outer membrane. Based on this, Vaara and Vaara [20] used the sensitization of *S.* Thypimurium to novobiocin as an indicator of outer membrane permeability changes in the presence of cationic agents. In a similar manner, we studied if the *S.* Thypimurium resistance to novobiocin was circumvented by growing bacteria in acidic pH condition. To this end, we determined CFU mL^-1^ at different times after exposure to novobiocin (see Methods). As expected, we observed that 0.15 μM novobiocin did not affect *S.* Thypimurium growth at neutral pH whereas at pH 4.7, the antibiotic reduced 90% of colony counts after 24 h of incubation (Figure 5). Taken together, our results suggest that low pH incubation modifies the outer membrane permeability, allowing the entry of MccJ25 and novobiocin into the cell.

As a mean of simulating internal macrophage conditions, antibiotic sensitivity assays were carried out in M9 medium without nutrient supplementation. However, we considered interesting to evaluate the low pH effect on the sensitivity of *S.* Thypimurium to MccJ25 and novobiocin when bacteria are cultured in a medium that allows bacterial growth. The *S.* Thypimurium viability upon antibiotic treatment was estimated by calculating CFU mL^-1^ after 24 h of incubation in M9 medium (pH 4.7) supplemented with 0.2% glucose, 0.2% casamino acids and 10 μM MgSO_4_. In fact, compared with the control (no antibiotic added), surviving bacteria were 0.0001 and 0.1% for cultures treated with MccJ25 and novobiocin, respectively (Data not shown). Since bacterial physiology is radically different in actively growing cultures compared with cultures in non-supplemented minimal medium, the observation of the low pH effect in both conditions strengthen the idea that low pH is a determinant feature in turning resistant bacteria to MccJ25 and novobiocin into sensitive ones.

In summary, these results present evidence that the previously reported resistance of *S.* Thypimurium to MccJ25 and novobiocin, produced by the inability of the antibiotics to penetrate the bacterial outer membrane [9,19], could be overcome when cells are exposed to low pH.

## Conclusions

In the present work we demonstrated that MccJ25 has an inhibitory effect on the intracellular replication of an *in vitro MccJ25-resistant* strain of *S.* Typhimurium. We suggest that the low pH of the macrophage environment is responsible for this effect, possibly by modifying the bacterial outer-membrane permeability.

From our results we can infer that several intracellular pathogenic strains that are naturally resistant to the antibiotic *in vitro* could be sensitive *in vivo* and that the action spectrum of MccJ25 may be broader than what *in vitro* studies suggested.

## Methods

### Bacterial strains and culture conditions

*S.* Typhimurium 14028s was obtained from the American Type Culture Collection. MC4100 *fhuA*::Km *E. coli* strain was supplied from the Dr. Salomon’ laboratory. Strains were grown on LB medium at 37°C. Kanamycin was added at a final concentration of 50 μg mL^-1^ for MC4100 *fhuA*::Km growth. For growth under low-iron conditions we used the Tris-buffered medium (T medium) without iron addition [21].

### MccJ25 effect on *S.* Typhimurium 14028s survival within macrophages

RAW 264.7 macrophages were infected with *S.* Typhimurium 14028s strain following the protocol previously described [10]. After infection, macrophages were washed three times with sterile PBS and incubated in fresh medium containing 100 μg mL^-1^ gentamycin without (control) or with 117.5 μM MccJ25. This concentration was selected based on the MccJ25 MIC for *S*. Typhimurium in the presence of (KFF)3K permeabilizing peptide [10]. At 0, 8, 18 and 24 h after MccJ25 treatment, macrophages were lysed with 0.2% Triton X-100 and the number of surviving bacteria was determined by subsequent plating on LB agar and CFU mL^-1^ count.

### MccJ25 effect on *S*. Typhimurium viability after replication within macrophages

*S.* Typhimurium cells were harvested from macrophages and then challenged with MccJ25 (117.5 μM). To this end, RAW 264.7 macrophages were infected with *S.* Typhimurium 14028s strain and 8 h post-infection were lysed as explained above. A fraction of the lysed macrophages (containing approximately 10^6^ mL^-1^ bacteria) was incubated with MccJ25, while another fraction with no antibiotic added served as control. Additionally, 10^6^ mL^-1^*S.* Typhimurium 14028s cells growing in LB medium were resuspended in 0.2% Triton X-100 and incubated with or without 117.5 μM MccJ25. After 6 h of incubation at 37°C, bacteria from within macrophages and those cultured in LB medium were diluted and CFU mL^-1^ was determined by plating on LB agar.

### Effect of low pH on sensitivity to MccJ25

In order to evaluate the pH influence on *S.* Typhimurium sensitivity to MccJ25 two assays were carried out. First, 10^6^ mL^-1^ bacteria were resuspended in M9 medium pH 7 or pH 4.7 (M9 acidified with HCl) and then supplemented with 117.5 μM MccJ25 (treated) or sterile water (control). After 0, 6, 8 and 24 h of incubation at 37°C, cells were plated on LB agar for CFU mL^-1^ determination.

As a second approach, we preincubated 10^6^ mL^-1^*S.* Typhimurium cells in M9 pH 7 or pH 4.7 for 0, 6 and 24 h at 37°C. At these time points, a 5-mL aliquot of each cell suspension was washed and resuspended in PBS (pH=7.4). A fraction of this suspension was treated with MccJ25 (117.5 μM) while another one with no MccJ25 added served as control. After 6 h of incubation at 37°C, CFU mL^-1^ was determined.

The sensitivity of MC4100 *fhuA*::Km to MccJ25 was determined by a spot-on-lawn test, as follows. Doubling dilutions of microcin solution (1 mg/mL) were spotted (10 μl) onto M9 plates at pH 7 or pH 4.7. Afterwards, 50 μl aliquots of a stationary phase culture of MC4100 *fhuA*::Km strain were mixed with 3 mL 0.6% agar and overlaid onto the plates. After overnight incubation, plates were examined for growth inhibition and the highest dilution with a clear halo of inhibition was considered as the MIC.

### Novobiocin sensitivity assay

Sensitivity of *S.* Typhimurium to novobiocin was evaluated by viable determination (CFU mL^-1^). Approximately 10^6^ mL^-1^ bacteria were resuspended in M9 either at pH 7 or pH 4.7. Then, cell suspensions were supplemented with novobiocin (0.15 μM) or sterile bidistilled water (control). After 0, 6 and 24 h of incubation at 37°C, CFU mL^-1^ was determined.

## Abbreviations

CFU: Colony-forming units; CPC: Cetylpyridinium chloride; E. coli: *Escherichia coli*; H: Hour; LB: Luria-Bertani medium; MccJ25: Microcin J25; MIC: Minimum inhibitory concentration; PBS: Phosphate buffered saline; RNAP: RNA polymerase; S. Typhimurium: *Salmonella enterica* serovar Typhimurium; T-medium: Tris-buffered medium.

## Competing interests

The authors declare that they have no competing interests.

## Authors’ contributions

MFP carried out the macrophage studies. NSC evaluated the effect of pH on the sensitivity to MccJ25. CA and RdeC participated in the design of the study. RNF helped to draft the manuscript. MAD and PAV conceived the study, and participated in its design, coordination and wrote the manuscript. All authors read and approved the final manuscript.
